# Heat stress dichotomy: long-term adaptation and acute shock in London domestic environments

**DOI:** 10.1098/rsta.2024.0567

**Published:** 2025-11-06

**Authors:** Maoran Sun, Jiayu Pan, Qunshan Zhao, Ronita Bardhan

**Affiliations:** ^1^ University of Cambridge, Cambridge, UK; ^2^ Department of Architecture, University of Cambridge, Cambridge, UK; ^3^ University of Glasgow, Glasgow, UK

**Keywords:** heatwave, indoor mini-heatwave, thermal stress, sensor data, summer indoor overheating

## Abstract

Europe is consistently experiencing hottest summers. Understanding people’s thermal comfort and stress and responses to heatwaves has become increasingly important. While much of the literature has recognized the overheating risks in the UK’s domestic housing stock, there remain short comings in analysing residents’ indoor heat exposure during heatwaves. This research aims to investigate the mini heatwaves occurring in domestic environments and to explore the factors influencing residents’ responses to heatwaves. A sensor-enhanced housing data survey was conducted in Southwark, London, over two summer months of 2023 during heatwave events. This study integrates outdoor weather data, sensor-measured high temporal-resolution indoor environmental conditions, the Index for Multiple Deprivation (IMD) and building features to analyse indoor heatwaves and thermal comfort. The article breaks ground by advancing existing discussions of urban heat stress, which typically focus on outdoor environments, by specifically examining indoor heat exposure intensities and the associated risks owing to vulnerability from asymmetry in adaptive capacities. In addition, the article aims to complement the current heatwave classifications based on the domestic heatwaves experienced by residents.

This article is part of the theme issue ‘Urban heat spreading above and below ground’.

## 1. Introduction

Global temperatures have been rising rapidly in recent years, with the UK experiencing its warmest recorded temperatures by a large margin [1,2]. Understanding people’s thermal comfort and stress and responses to heatwaves has become increasingly important [3]. While much of the literature has recognized the overheating risks in the UK’s domestic housing stock [4,5], there remains potential in analysing residents’ indoor heat exposure during heatwaves. Homes can amplify or mitigate heat differently depending on factors such as building design, insulation and ventilation, making it essential to shift focus towards monitoring and analysing indoor thermal conditions. This knowledge is vital for developing targeted strategies to protect vulnerable populations and improve the resilience of residential buildings to extreme heat.

While the study on heatwaves and their impacts has gained interest [6–8], this area of research remains underexplored, particularly in the context of indoor environments [9]. Most studies rely on meteorological data or simulations [10], which, while valuable, often fail to capture how heatwaves affect indoor spaces and occupant thermal comfort in different house types and households. Sensor-collected data offer a unique opportunity by providing high spatial resolution, real-time insights into indoor temperature fluctuations and thermal comfort levels during extreme heat events. However, the potential of such data remains underutilized, with limited research focusing on how indoor conditions respond to outdoor heatwaves, especially in residential settings.

This study aims to offer an understanding of indoor thermal comfort during heatwaves and contributes to the development of more resilient housing strategies in the face of a warming climate. This research investigates the phenomenon of indoor mini-heatwaves in domestic environments affected by outdoor heatwaves. Indoor mini-heatwaves refer to short but continuous periods of thermal discomfort caused by elevated temperatures within the indoor environment. Finally, we explore the housing design factors influencing the indoor thermal environment.

To better understand the indoor thermal environment, sensor-enhanced housing survey data [11] were collected in Southwark, London, over two months during the summer of 2023, which faced a national heatwave. This study integrates multiple household-level datasets that inform the outdoor and indoor environmental conditions, building design features and socio-economic data that serve as indicators of heat stress adaptation options available in the households. Weather data collected from the local weather station was used to develop the outdoor conditions and in detecting heatwave periods. The indoor conditions were collected through high-temporal resolution environmental sensors. The socio-economic and demographic conditions were captured through the Index for Multiple Deprivation (IMD), which combines multiple indicators of deprivation that enable adaptation to heat stress. These indicators include income, employment, health, education, crime, barriers to housing and living environment. The energy performance certificate (EPC) and building design features were used to evaluate the condition of the dwellings’ heat resiliency. These parameters were then comprehensively used to analyse indoor mini-heatwaves and thermal comfort.

A multi-level understanding of the domestic thermal environment is established with the sensor data. A psychrometric chart was constructed to analyse the thermal environment within domestic settings during summer in London. This chart provides insights into the relationship between temperature, humidity and thermal comfort, offering a comprehensive understanding of indoor climate conditions. In addition, an algorithm was implemented to identify short-term heat spikes within buildings, enabling a detailed examination of how mini-heatwaves vary according to building characteristics and socio-economic status. To further investigate the contributing factors to rising indoor temperatures, a regression analysis was conducted. This analysis sheds light on the influence of variables such as building condition, energy efficiency and socio-economic factors on indoor thermal performance, providing valuable insights for improving building design and policy interventions.

This article breaks ground by advancing existing discussions of urban heat, which typically focus on outdoor environments, by specifically examining indoor heat exposure intensities and the associated risks owing to vulnerability from asymmetry in adaptive capacities.

## 2. Background

### (a) Definition of heatwave and mini-heatwave

The classification of heatwaves remains inconsistent and lacks a universally accepted definition, posing challenges for researchers and policymakers [12]. While various meteorological organizations and studies have proposed criteria—such as temperature thresholds [13], duration [13,14] and regional variability [15]—there is no standardized framework that applies globally or even nationally.

In the UK, a heatwave is officially declared when a location records at least three consecutive days with daily maximum temperatures reaching or surpassing a specific heatwave threshold [16]. This threshold is not standardized nationwide but instead varies by county, accounting for regional climatic variations. For London, the threshold is set at 28°C, reflecting the city’s unique climatic conditions and urban heat characteristics.

The existing definition focuses solely on outdoor temperatures, which do not always accurately reflect conditions within domestic environments. As heatwaves become more frequent and intense owing to climate change, understanding how these extreme events affect indoor spaces is critical.

Although research on indoor overheating has expanded rapidly [17,18], the field still lacks a common benchmark for what constitutes an ‘overheat’ event [19]. Without at least a nationally or regionally standardized definition of indoor heatwaves, it remains impossible to compare studies, identify at-risk dwellings or craft targeted policy. Establishing such a definition is therefore a critical next step for overheating research and practice.

We introduce the concept of an indoor ‘mini-heatwave’—an analogue to the UK’s outdoor heatwave definition but tailored to interior conditions. Unlike the outdoor criterion, which requires threshold temperatures to persist for three consecutive days, an indoor mini-heatwave may be triggered over a shorter duration. An advantage of our method is its flexibility—it allows the duration threshold to be adjusted based on specific research objectives or data resolution, making it adaptable to different contexts or comfort standards. We initially set the minimum duration threshold to 10 min and conducted a sensitivity analysis to examine how varying this parameter influences the detection results.

Thresholds should be set nationally or regionally. For England, the Heatwave Plan (2011) already flags 26°C as the upper limit for safe indoor conditions [20,21]. This cut-off is reinforced by evidence that mortality rises once indoor temperatures exceed 26°C [22]. Accordingly, we adopt 26°C as the working threshold for defining an indoor mini-heatwave in this study.

### (b) Heatwave and its influence on domestic environments

Heatwaves have profound implications for domestic environments, affecting human health [23], energy consumption [24] and socio-economic equity [25]. Research highlights that elevated outdoor and indoor temperatures significantly impair human health and productivity, and increase cooling energy demand, leading to higher peak electricity loads [26] and heat-related mortality [27,28]. Deviations from optimal indoor temperatures disrupt human thermoregulation, causing discomfort, reduced productivity and dissatisfaction [29,30]. Prolonged exposure to extreme heat exacerbates cardiovascular, respiratory and thermoregulatory issues, such as strokes and exhaustion, while also contributing to cognitive and mental health disorders [31,32]. These effects are particularly pronounced in vulnerable populations, including the elderly, those with pre-existing health conditions, and individuals lacking financial or public support [25].

Despite the significant implications of heatwaves and indoor overheating for human health, energy consumption and social equity, the driving factors behind these phenomena and their relationship with outdoor weather conditions have not received sufficient attention in the literature. While the prediction of indoor building temperature has been extensively studied (e.g. [33–36]), there remain limitations in understanding the underlying link between outdoor weather patterns and indoor thermal conditions. This highlights the need for further research to explore the complex interplay between external climatic factors and indoor overheating, particularly in the context of rising global temperatures and the increasing frequency of extreme heat events.

## 3. Study area and data

### (a) Study area

**Figure 1.**
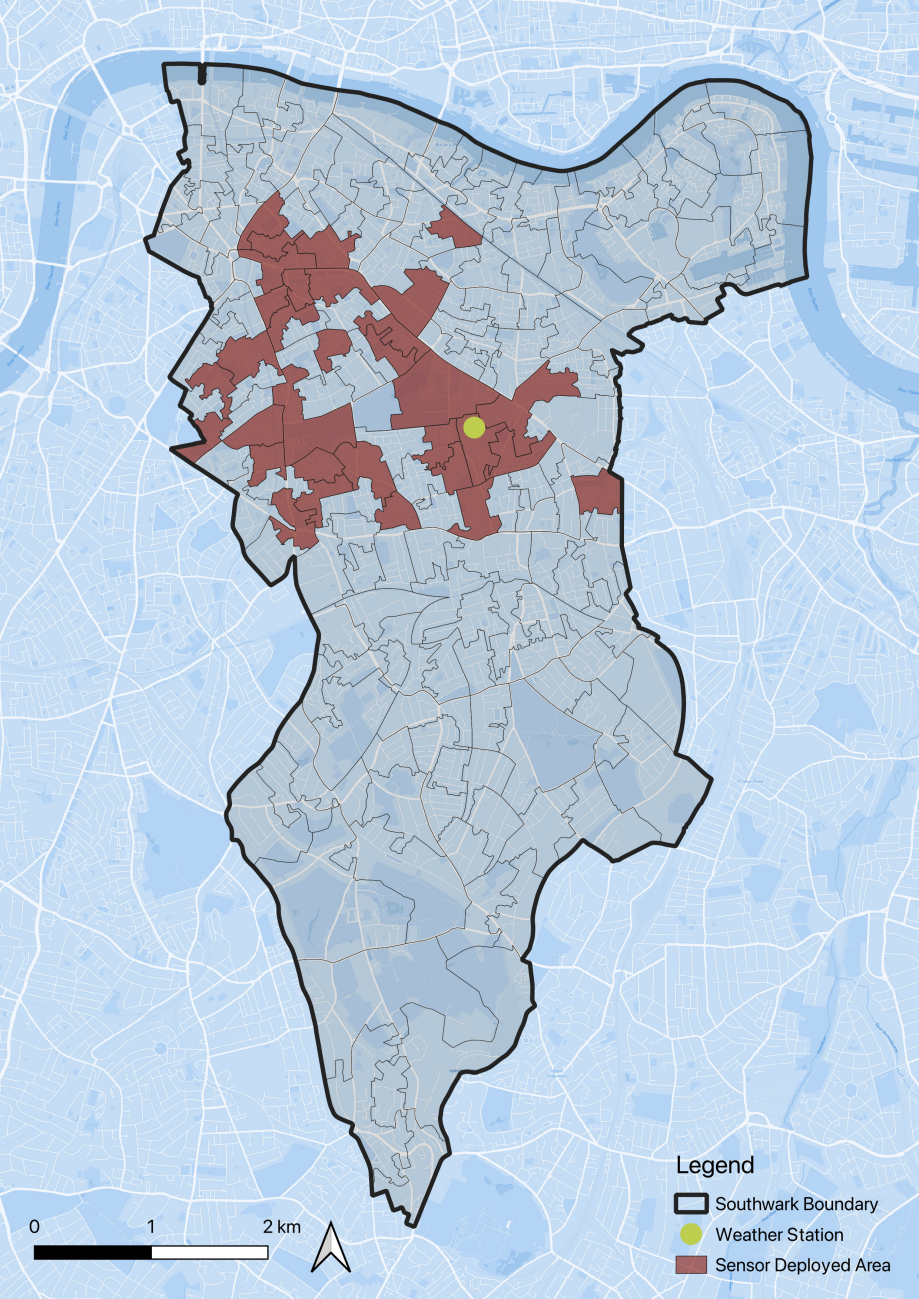
Study area.

The study area selected for this research is Southwark, London (see figure 1 ). London is defined as a temperate oceanic climate, based on the Köppen climate classification. Southwark is a borough of particular significance owing to its unique characteristics and challenges. In 2021, it was recorded as one of the hottest locations in the UK, making it a critical area for studying heat-related issues. Based on weather reports collected during 2012–2021 [37], July is the hottest month for Southwark, with maximum, minimum and mean temperatures of 24°C, 14°C and 19°C, respectively. The borough is ranked as the 12th most deprived area in London according to the IMD [38], underscoring the vulnerability of its residents to summer overheating.

### (b) Data

#### (i) Indoor sensor data

The primary dataset for this study consists of sensor-collected data gathered from 40 homes in Southwark, London. Indoor environmental conditions were monitored using Smart Citizen Kit sensors [39], which recorded *air temperature, relative humidity, air pressure, air quality, noise level and ambient light at 1 min intervals*. The detailed sensor specifications can be found at the Smart Citizen website.^1^ Participants were instructed to install the sensors in their bedroom, positioned away from direct sunlight whenever possible. This was done to minimize bias owing to localized solar gain or radiative effects. The data collection period extended from July 28 to 26 September 2023, capturing indoor conditions during one of the hottest summers on record in the UK. We further filtered the dataset to include records from August 2 to September 13, ensuring the most consistent and overlapping data coverage across different sensors. This high-temporal resolution dataset provides a detailed understanding of indoor thermal environments and their response to external heatwaves.

#### (ii) Outdoor weather station data

Outdoor weather measurements were sourced from a nearby local weather station, LIMBO [40]. This station provided continuous climate data, including air temperature, relative humidity and wind speed, recorded at 5 min intervals. The weather station is located within the study area borough, surrounded primarily by low- to mid-rise residential buildings. As such, it is considered broadly representative of the residential neighbourhoods in the city. The high-frequency data from LIMBO offers a comprehensive and precise representation of outdoor conditions, enabling a detailed comparison with indoor environmental data collected during the same period.

#### (iii) Household attributes

In addition to the air temperature and relative humidity data, this study also gathered detailed information on building characteristics and household profiles. A survey was conducted for each participating household, capturing self-report housing condition, IMD quintile, housing type and tenure types. Furthermore, EPC data was collected using the home addresses, providing additional insights into the energy efficiency and thermal performance of the buildings. This multi-faceted dataset enables a comprehensive analysis of how indoor thermal conditions are influenced by both environmental and structural factors, as well as occupant behaviour and socio-economic contexts.

## 4. Methodology

### (a) Indoor thermal comfort

To evaluate and represent thermal comfort conditions, a psychrometric chart and a heat index were employed as tools to analyse the relationship between air temperature, relative humidity and other thermodynamic properties. The psychrometric chart offers a comprehensive visualization of the thermal environment, enabling the identification of comfort zones based on established standards such as ASHRAE Standard 55 or other relevant guidelines. This method is particularly effective for assessing indoor thermal conditions and interpreting the interplay between temperature, humidity and human comfort. The heat index is a widely used metric that combines air temperature and relative humidity to estimate perceived temperature and human thermal stress. While originally developed for outdoor conditions, it can serve as a simplified indicator of indoor thermal discomfort during heatwaves.

Environmental parameters including dry-bulb temperature (°C), relative humidity (%) and air velocity (m s^−1^) were used in the calculation. The dry-bulb temperature and relative humidity are derived from the sensor reading. Air velocity is set to 0.1 m s^−1^ as it is in the indoor environment [41]. These data points were first plotted on the psychrometric chart, where the *x*-axis represents the dry-bulb temperature and the *y*-axis represents the humidity ratio (moisture content in the air). Curves of constant relative humidity and enthalpy were used to interpret the thermal conditions and provide a clear understanding of the environmental state. The chart is plotted using the psychrochart Python library. A heat index was calculated further to provide a more intuitive assessment of the thermal comfort based on equation (4.1) [42]. Note that in equation (4.1), the temperature $T_{F}$ is a converted temperature reading in Fahrenheit from the sensor reading, which is in Celsius.


(4.1) $$\begin{array}{rl} HI= & -42.379+2.04901523T_{F}+10.14333127RH-0.22475541T_{F}RH\\ & -6.83783\times 10^{-3}T_{F}^{2}-5.481717\times 10^{-2}RH^{2}\\ & +1.22874\times 10^{-3}T_{F}^{2}RH+8.5282\times 10^{-4}T_{F}RH^{2}\\ & -1.99\times 10^{-6}T_{F}^{2}RH^{2}, \end{array}$$

where: *HI* is the heat index, $T_{F}$ represents the temperature in Fahrenheit converted from the sensor reading and RH denotes the relative humidity.

The use of the psychrometric chart and heat index in this study offered several advantages. The psychrometric chart provided an intuitive and visually accessible representation of complex thermodynamic relationships, enabling rapid identification of comfort zones and deviations under varying indoor conditions. Complementing this, the heat index offered a straightforward yet effective means of estimating perceived thermal discomfort during heatwaves by accounting for both temperature and humidity. Together, these methods allowed for a comprehensive and communicable assessment of indoor thermal comfort levels.

### (b) Detection of short heat strike in domestic environments

**Figure 2.**
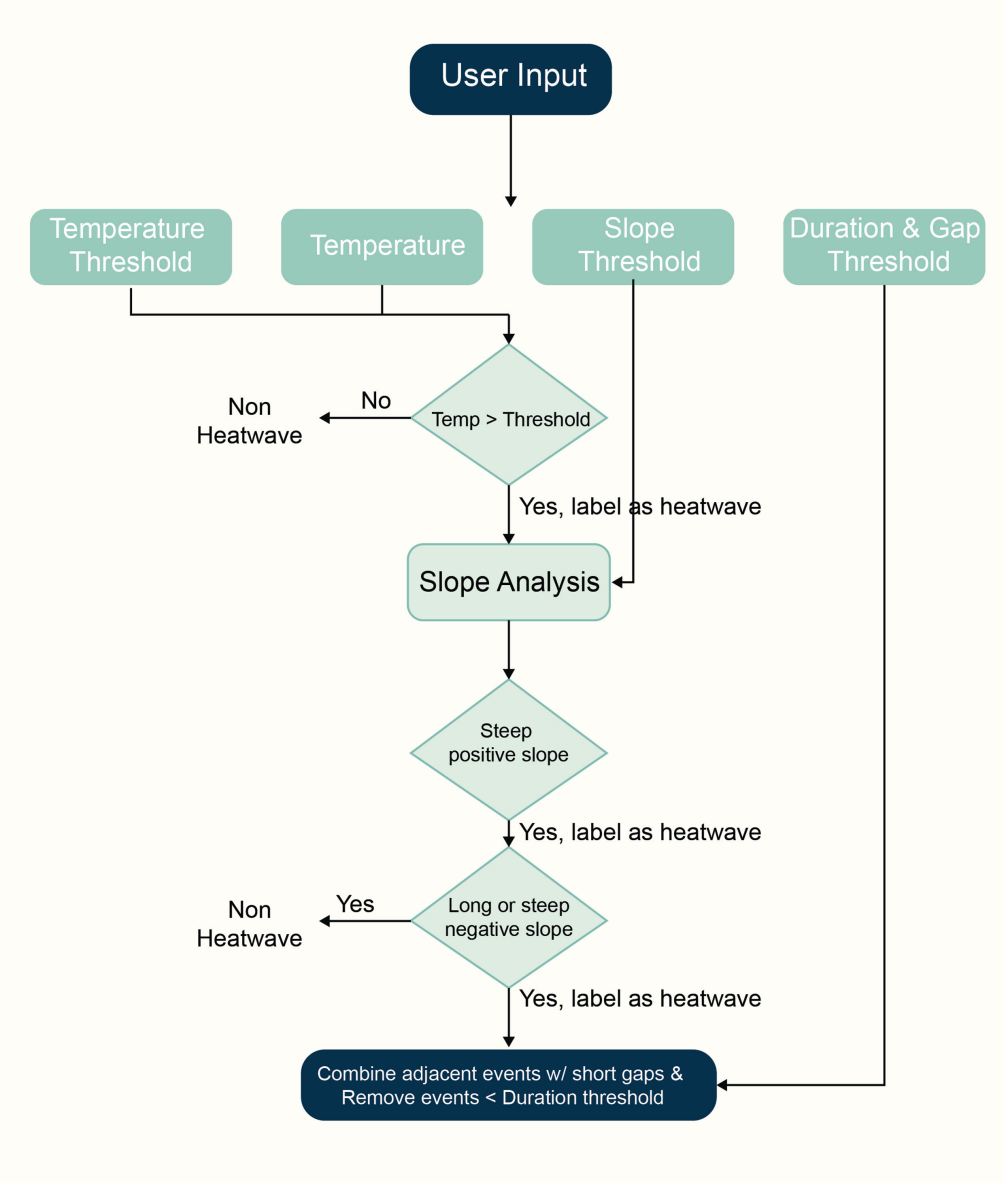
Mini-heatwave detection illustration.

In addition to examining indoor thermal comfort at individual data points, this study also investigates the continuous fluctuations in indoor thermal comfort and the mini-heatwaves induced by outdoor weather conditions. To achieve this, we developed and implemented a specialized algorithm called the heatwave-detector, based on the Firefinder algorithm [43]. The Firefinder algorithm initially was implemented to detect cooking events using stove temperature monitors to indicate usage. We adapted the method to identify and further analyse patterns in indoor temperature data that signify continuous mini-heatwaves as shown in figure 2.

The heatwave-detector algorithm identifies short-term indoor overheating events, referred to as mini-heatwaves, using user-defined inputs: time-series temperature data, a temperature threshold, a minimum event duration threshold, a threshold for temperature increase rate and minimal gaps between events.

The algorithm begins by flagging all data points where the temperature exceeds the specified threshold as potential mini-heatwave candidates. To improve robustness against sensor noise or erroneous readings, it then applies additional validation steps.

A slope-based analysis is performed to capture the dynamics of temperature change. Specifically:

(i) Data points with a rapidly increasing temperature (greater than $2^{\circ }$C min^–1^) are included in the event, even if the temperature has not yet reached the threshold. This allows the algorithm to capture the onset of heating events.
(ii) Conversely, data points exhibiting steep negative slopes or sustained cooling trends are excluded to reduce false positives caused by post-peak cooling.

In the final step, the algorithm applies a smoothing procedure based on the user-defined duration threshold. Temporally adjacent candidate events separated by short gaps are merged, and events shorter than the minimum duration are discarded. This ensures that only meaningful and persistent indoor heat events are retained.

This method offers several key advantages. First, it is highly flexible: both the temperature and duration thresholds can be adjusted to suit different research contexts, sensor resolutions or definitions of thermal discomfort. Second, by incorporating slope-based logic, the algorithm captures both the onset and decline of thermal events more accurately than threshold-based methods alone. Finally, its smoothing and noise-handling steps make it robust to short-term fluctuations and sensor irregularities, improving the reliability of event detection in real-world indoor environments.

### (c) Contributing factors to rising indoor temperature

Understanding the contributing factors to high indoor temperatures is crucial for proposing and implementing effective design strategies and policy interventions. To achieve this, a multiple linear regression model was employed to estimate the effects of building characteristics, outdoor weather conditions and the socio-economic status of homeowners on indoor temperature. The selection of independent variables was guided by both data availability and existing literature. Prior studies [44,45] commonly include variables such as tenure, house type and outdoor temperature when modelling indoor temperature. To match the temporal resolution of outdoor temperature (5 min), the indoor temperature is aggregated to 5 min resolution by taking the average. The primary hypothesis of the model is formalized in equation (4.2).


(4.2) $$\begin{array}{r} TempIndoor=\beta_{0}+\beta_{1}\cdot TempOutdoor+\beta_{2}\cdot HouseConditionSocioEconomic\\ +\beta_{3}\cdot Tenure+\beta_{4}\cdot HouseType+\beta_{5}\cdot LaggedTempOutdoor, \end{array}$$

where: $\beta_{0}$ represents the intercept term and $\beta_{1}$, $\beta_{2}$, $\beta_{3}$, $\beta_{4}$ and $\beta_{5}$ denote the coefficients associated with the independent variables.

The regression model was estimated using the ordinary least-squares method. The statistical significance of the coefficients was evaluated using *p*-values, with a threshold of 0.01 employed to determine significance. The overall explanatory power of the model was assessed using the R-squared and adjusted R-squared metrics.

The primary inputs to the model consist of three distinct types of variables. First, continuous variables were included to capture continuous measurements. These variables encompass outdoor temperature and lagged outdoor temperature.

Second, categorical variables were incorporated using dummy encoding to facilitate their inclusion in the regression analysis. Specifically:

— House type was operationalized through three dummy variables: ‘flat/apartment (high-rise on a low floor, or low-rise)’, ‘flat/apartment (high-rise, on a mid to high floor)’ and ‘terraced house’.
— Tenure type was represented by five dummy variables: ‘homeowner with mortgage’, ‘renting from a housing association/housing co-operative or charitable trust’, ‘renting from a local authority/council’, ‘renting from a private landlord’ and ‘shared ownership’.

Third, ordinal features are incorporated to the model. The IMD is an ordinal variable. In addition to IMD, ordinal encoding was applied to variables with inherent ranking or order. Specifically:

— EPC rating was encoded on a scale from 0 to 3, where 0 represents the most energy-efficient rating (B) and 3 represents the least efficient rating (E).
— Self-rated building condition was assigned values ranging from 0 to 3, with 0 indicating the worst condition and 3 representing the best condition.

This encoding scheme ensures that the ordinal nature of these variables is preserved while allowing them to be incorporated into the regression model effectively.

## 5. Results

### (a) Descriptive statistics

In this study, a total of 40 sensors were deployed across households in Southwark, London. During the data pre-processing phase, one sensor was excluded owing to abnormal readings, which were consistently and significantly higher than those of other sensors. Among the remaining sensors, 33 households had associated EPC data, and all households had corresponding IMD data. Table 1 lists the distribution of the available house characteristics (EPC ratings, IMD scores, tenure, house type, self-report house condition) across the sampled households. The table demonstrates that the distribution of both EPC ratings and IMD scores is relatively balanced, indicating a representative sample that captures a wide range of building energy efficiencies and socio-economic conditions.

**Table 1.** House characteristics distribution.

	category	count
EPC	B	12
	C	9
	D	8
	E	4
IMD	1 (more deprived)	16
	2 (less deprived)	23
tenure	homeowner with mortgage	14
	renting from a local authority/council	12
	renting from a housing association/housing cooperative or charitable trust	6
	renting from a private landlord	5
	shared ownership	2
house type	flat/apartment (high-rise, on a mid to high floor)	16
	flat/apartment (high-rise on a low floor, or low-rise)	14
	terraced house	9
self-report house condition	not new but in reasonable condition	19
	new and/or in good condition	13
	poor condition, in need of repair	5
	very poor condition, in need of urgent repair	2

During the data collection period, two outdoor heatwaves were defined based on the heatwave definition of the UK Met Office, occurring from August 22–24 and September 4–11, respectively. For the August heatwave, the maximum, mean and minimum outdoor temperatures were 29.8°C, 22.3°C and 15.9°C, respectively. For the September heatwave, the maximum, mean and minimum outdoor temperatures were 34.7°C, 24.0°C and 15.9°C, respectively.

**Figure 3.**
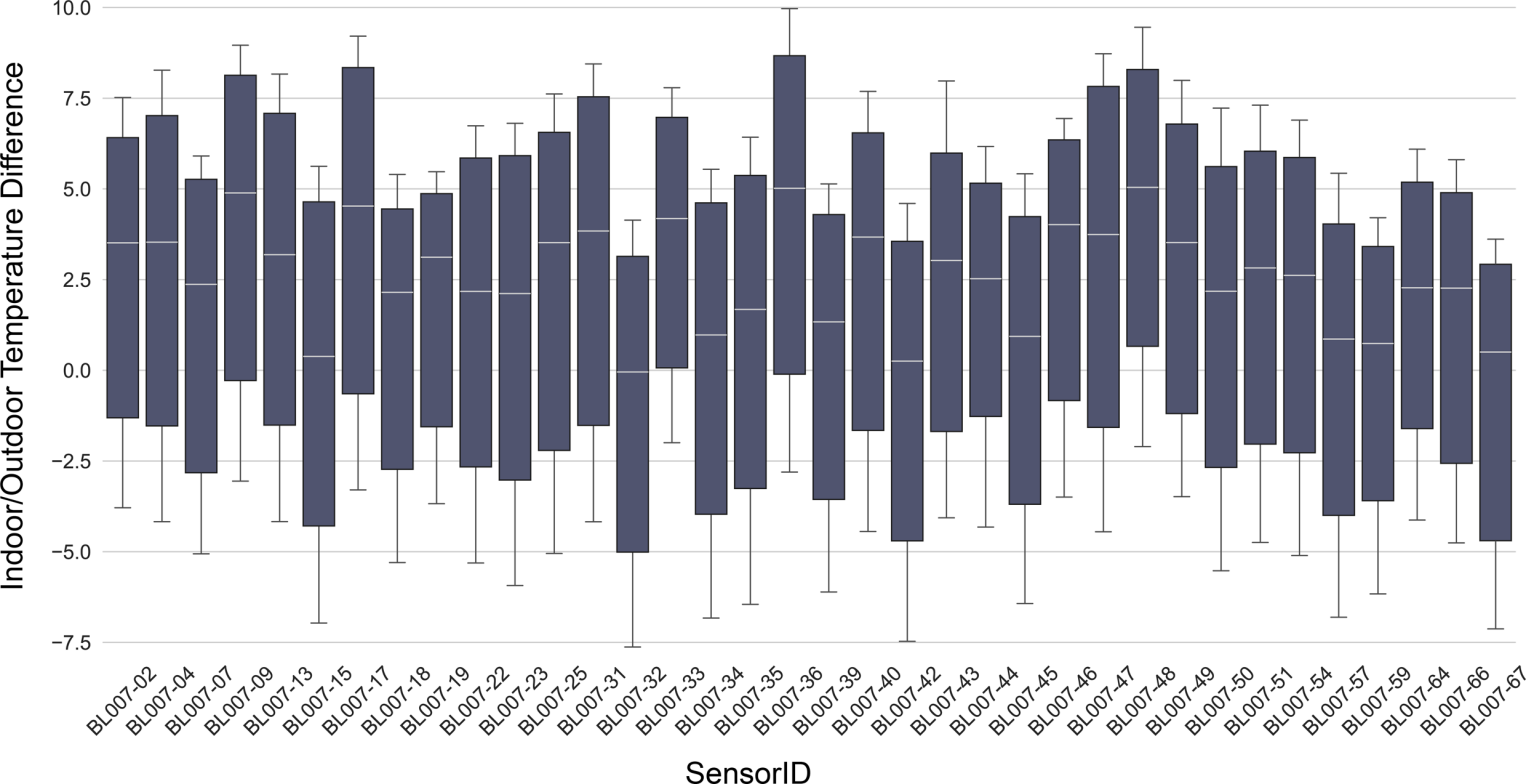
Variations of hourly indoor/outdoor temperature difference by household during Sep 4 to Sep 11 heatwave.

To illustrate inter-household variability during extreme heat conditions, we computed hourly averaged indoor–outdoor temperature differences across all sensors during the two outdoor heatwave periods. We then plotted boxplots of these hourly values for every sensor in figure 3, allowing us to visualize the variations of indoor and outdoor temperature differences at each house. Across the households, the maximum mean temperature difference is 4.3°C, with the median and minimum being 1.7°C and −0.9°C. This method ensures that indoor temperature patterns are compared over a consistent time window, allowing us to highlight how different homes and building types respond to external heat conditions.

### (b) Indoor thermal comfort

Using the data collected from indoor sensors, we projected the thermal comfort level of each minute-by-minute sample on to a psychrometric chart and calculated the heat index.

**Figure 4.**
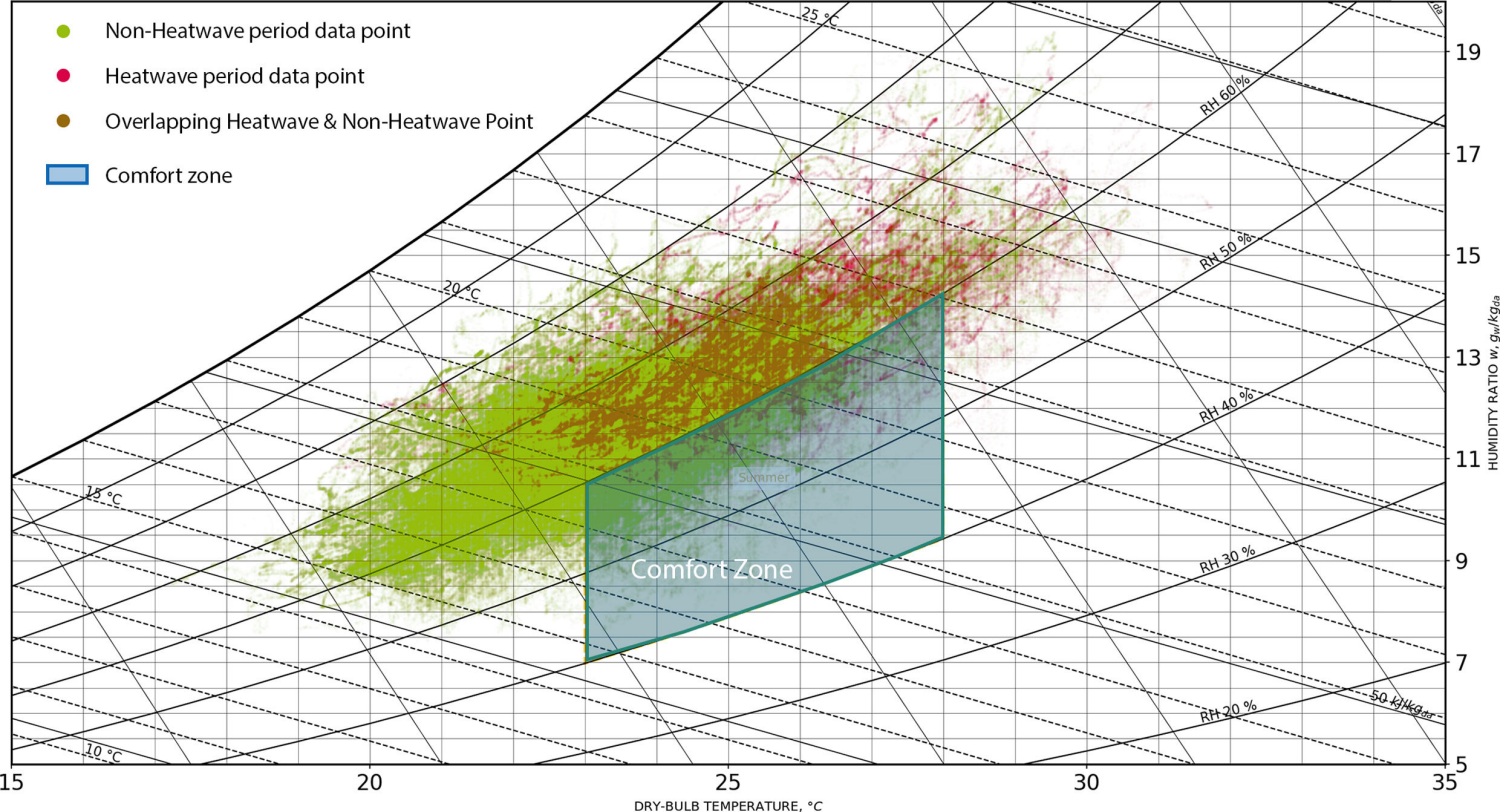
Psychrometric chart showing indoor thermal conditions. Each dot represents a data point, with its position indicating the corresponding temperature and relative humidity. Green dots represent data from non-heatwave periods, while red dots represent data from heatwave periods. Overlapping points may appear as mixed colours (orange) owing to the visual blending of red and green.

As shown in the psychrometric chart in figure 4, each dot on the chart represents an individual data sample, illustrating the relationship between temperature and humidity at that specific moment. The red dots represent the data sample during the outdoor heatwaves (August 22–24 and September 4–10). The green dots show the data sample during a non-heatwave. The thermal comfort zone, as defined by the ASHRAE Standard 55, is outlined on the chart. This zone represents the range of temperature and humidity levels within which most occupants are expected to feel comfortable, taking into account factors such as clothing insulation and metabolic rate.

As depicted in the figure 4, the majority of the plotted data points fall outside the acceptable limits of the thermal comfort zone based on the ASHRAE definition. This indicates that residents were exposed to conditions of excessive heat and humidity, leading to potential discomfort. Moreover, the non-heatwave periods (green) have more data points in the comfort zone.

The heat-index analysis yields conclusions consistent with those derived from the psychrometric chart. We first computed the absolute heat-index values and then categorized them into standard comfort levels following the National Weather Service classification (converted from Fahrenheit to Celsius) [46]: less than 26.7°C (comfortable), 26.7–32.2°C (caution), 32.2–39.4°C (extreme caution), 39.4–51.1°C (danger) and greater than 124°C (extreme danger). During the study period, the indoor heat-index values predominantly fell within the comfortable, caution and extreme caution categories, with the majority of readings classified as comfortable.

To further investigate the temporal dynamics, we compared comfort level distributions during and outside heatwave events. During outdoor heatwave periods, 53% of the indoor measurements remained in the comfortable category, 45% fell under caution and 2% under extreme caution. By contrast, outside heatwave periods, the proportion of time spent in the comfortable range increased significantly to 85%, with 13% in caution and only 2% in extreme caution.

The findings highlight the challenges faced by households in maintaining comfortable indoor environments during periods of extreme outdoor heat, underscoring the need for effective strategies to improve thermal resilience in residential settings.

### (c) Detection of short heat strike in domestic environments

Using the sensor data, we applied the heatwave-detector algorithm with a threshold of 10 min duration to identify mini-heatwave events within the indoor environment.

Following detection, of the 39 households monitored, 26 experienced at least one instance of indoor temperatures reaching mini-heatwave thresholds during the observation period. For the households that have mini heatwaves detected, we identified an average of 5.35 mini-heatwave events per household—a significantly higher number compared with the outdoor heatwaves recorded during the same period.

**Figure 5.**
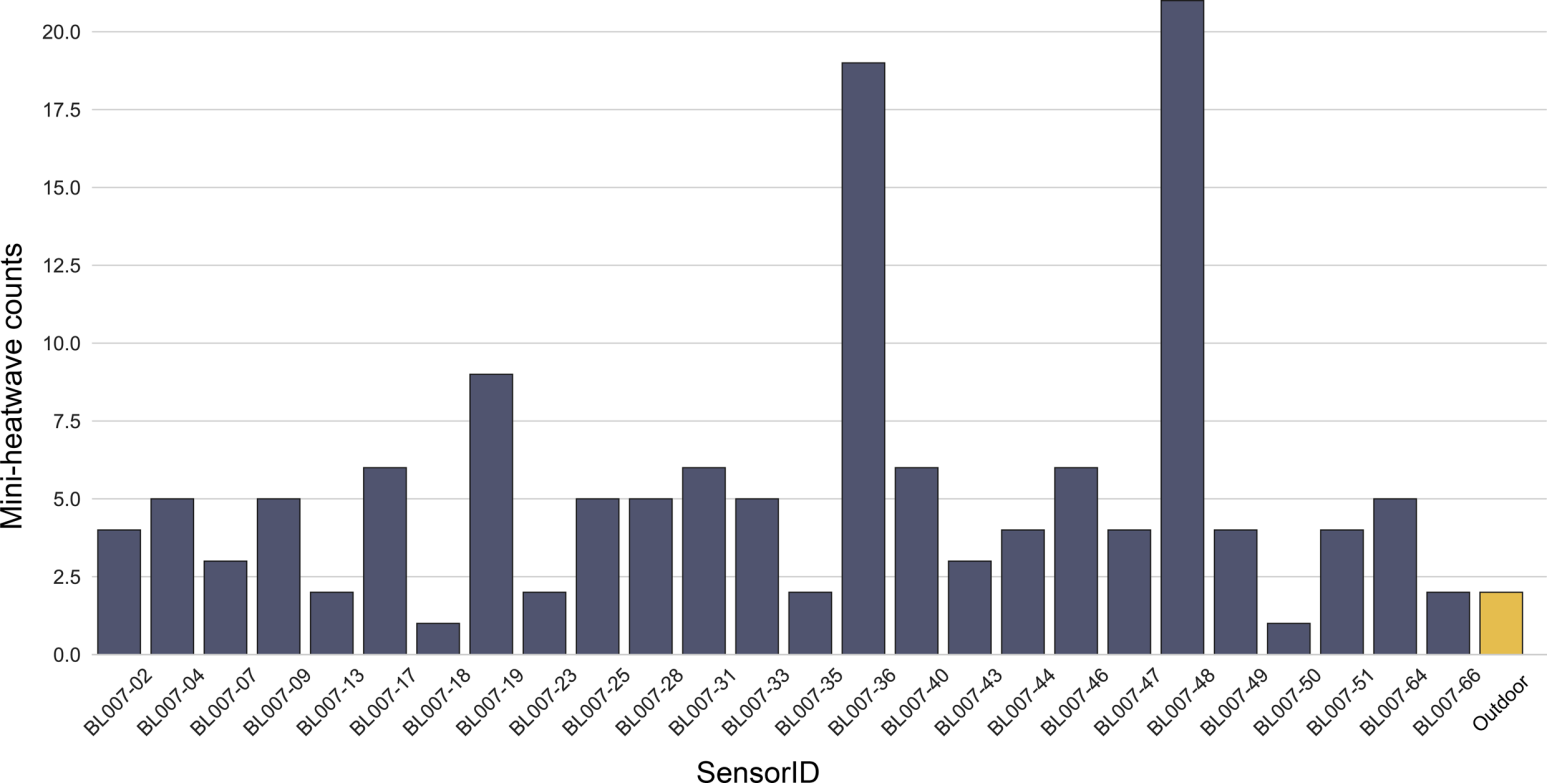
Mini-heatwave counts by household.

Figure 5 illustrates the distribution of detected mini-heatwaves across individual sensors. The results reveal significant variability, with the most affected household experiencing 21 mini-heatwave events. The yellow bar on the right-hand side of the figure represents the outdoor heatwave count. During the research period, only two outdoor heatwaves were detected.

Table 2 lists more of the mean count of mini-heatwave events per household, stratified by EPC rating, IMD and self-rated house condition. The analysis by EPC rating shows that households with an EPC band D exhibit the lowest average number of mini-heatwave events, with 3.5 events per house. By contrast, households in other EPC bands (e.g. B, C and E) show a higher and relatively consistent average of approximately six events per house. This suggests that buildings with moderate energy efficiency (EPC band D) may be less prone to overheating compared with those in other bands.

**Table 2.** Mini-heatwave counts by EPC, IMD and self-rated house condition.

	category	count
EPC	B	5.67
	C	6.33
	D	3.50
	E	6.00
IMD	1	3.88
	2	6.00
house condition	very poor condition, in need of urgent repair	4.50
	poor condition, in need of repair	5.00
	not new but in reasonable condition	5.62
	new and/or in good condition	5.25

When examining the relationship between deprivation (IMD) and mini-heatwaves, a clear pattern emerges. Households in the most deprived areas (IMD = 1) experience fewer mini-heatwave events, with an average of 3.88 events per house. By contrast, households in less deprived areas (higher IMD values) report an average of six events per house. This indicates that socio-economic status, as reflected by IMD, plays a significant role in shaping indoor thermal conditions, with less deprived areas being more susceptible to mini-heatwaves.

A similar trend is observed for the self-rated house condition. Buildings rated as being in poorer condition generally experience fewer mini-heatwave events, while those in better condition report higher counts. This finding aligns with the broader theme that well-maintained buildings, despite their advantages of good physical condition and fewer heating requirements during winters, may be more prone to overheating during summer months.

**Figure 6.**
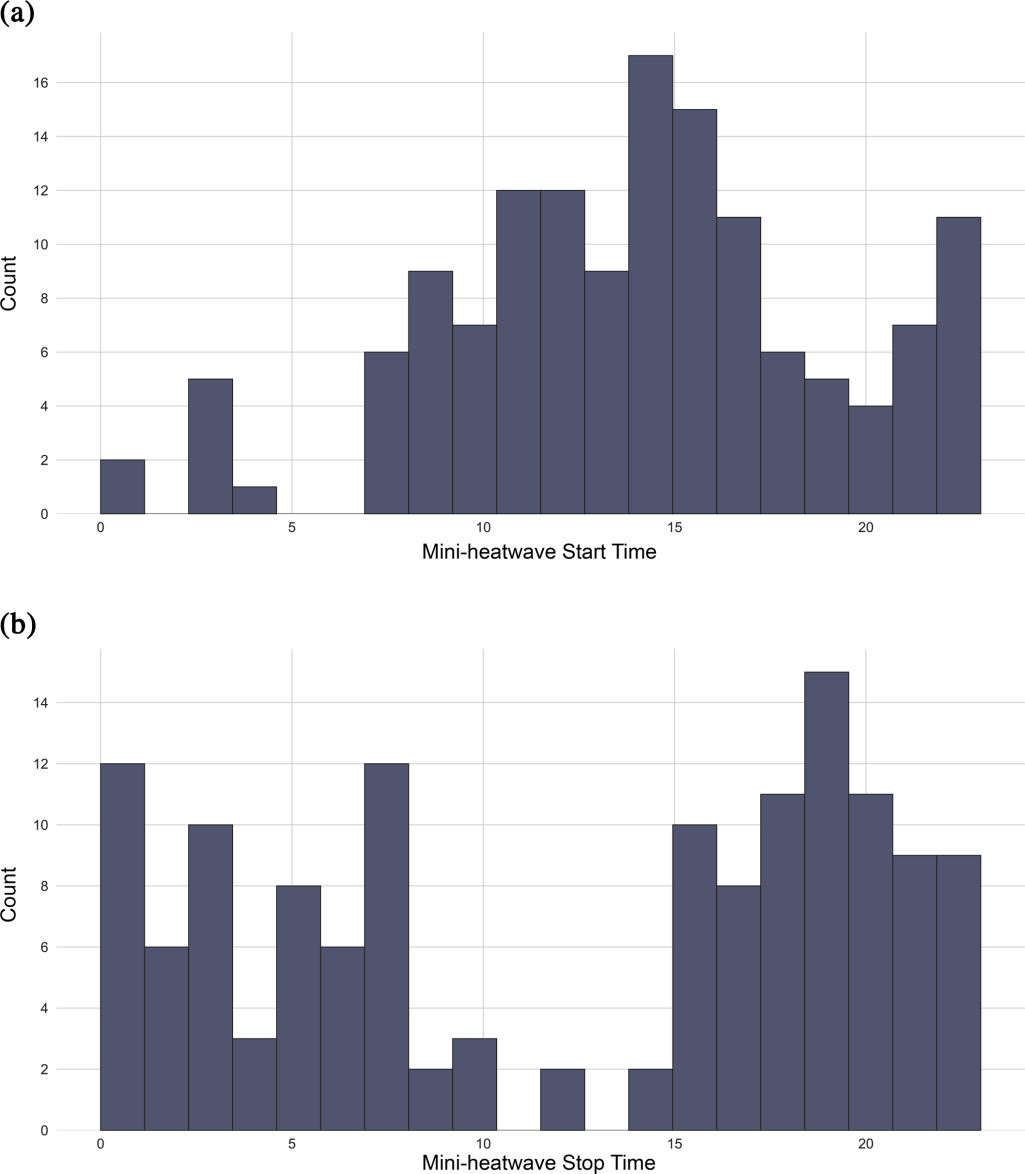
Histogram of mini-heatwave event start and stop times based on hour of day (0–23, in 24 h format). (a) Mini-heatwave start time (hour of day). (b) Mini-heatwave stop time (hour of day).

The majority of these indoor mini-heatwaves lasted approximately 477 min and predominantly occurred during the afternoon, extending into the night. Figure 6*a* reveals that most mini-heatwaves begin at approximately 15.00, aligning with peak outdoor temperatures. However, note that a considerable number of mini-heatwaves also initiate after 21.00, indicating that the delayed rise in indoor temperatures may be driven by persisting outdoor heat stress and residual heat stored in building materials. The termination of mini-heatwaves typically occurs at approximately 20.00, with a relatively stable number of events continuing from 20.00 to 1.00 (see figure 6b). This pattern underscores the persistence of indoor heat stress long after outdoor temperatures have subsided, highlighting the lag effect of heat accumulation within buildings. These findings emphasize the need to address indoor thermal conditions, particularly during evening and nighttime hours, when occupants are more likely to be at home and vulnerable to prolonged heat exposure.

We further conducted a sensitivity analysis by varying the duration threshold for detecting indoor mini-heatwaves. In addition to the previously used 10 min threshold, we tested thresholds of 30 min, 60 min and 120 min to capture more persistent overheating events and to align with the commonly available data resolution of 1 h.

We compared the number of households affected, the median count of mini-heatwave events per household, and the median duration of those events. Table 3 lists the comparison results. For thresholds of 10, 30 and 60 min, 26 households experienced at least one mini-heatwave. At the 120 min threshold, this number slightly decreased to 24 households.

**Table 3.** Sensitivity analysis for mini-heatwave duration threshold.

mini-heatwave duration threshold (min)	10	30	60	120
number of households affected	26	26	26	24
median count of mini-heatwave per house	4	4	4	4
median duration of mini-heatwave events	426	447	474	504.5

As expected, increasing the threshold duration led to longer median mini-heatwave durations: 426, 447, 474 and 504.5 min for 10, 30, 60 and 120 min thresholds, respectively. The median number of events per household remained consistent at 4.0 across all threshold values. However, we do observe a decrease in the mean number of events (5.1, 4.9, 4.4 and 4.3), which indicates that some shorter events are being filtered out as the threshold increases. The stability in the median suggests that these shorter events were not widespread across all households, and that most households continued to experience a similar number of persistent overheating events.

According to the UK’s official heatwave definition, only two heatwaves occurred during the entire data collection period. However, in most households, we detected indoor mini-heatwaves that overlapped with these outdoor heatwaves. In addition, we observed a lag effect in the indoor heatwaves, with most indoor events continuing even after outdoor temperatures begin to decline in the evening or nighttime hours. This delay underscores the capacity of buildings to retain heat, prolonging exposure to uncomfortable conditions even after outdoor temperatures have returned to normal levels. These findings emphasize the need to consider indoor thermal dynamics when assessing the effects of heatwaves and developing strategies to mitigate their effects on residents.

### (d) Contributing factors to rising indoor temperature

Five models were implemented to test the relationship between independent variables and the indoor temperature (5 min average). The base model (Model 1) only takes outdoor temperature, self-report house condition, EPC rating and IMD as input. Models 2 and 3 added tenure and house type dummy variable, respectively. Model 4 includes all of the above variables. Model 5 adds one more 6 h lagged outdoor temperature variable.

The regression analysis revealed significant relationships between indoor temperature and the independent variables, including building characteristics, outdoor weather conditions and socio-economic status of homeowners.

The overall model fit was assessed using the R-squared and adjusted R-squared values. The R-squared value of 0.33 indicates that approximately 33% of the variability in indoor temperature is explained by the independent variables included in the model. This result achieves better performance to a similar study [44].

The regression coefficients for each independent variable in four models are listed in table 4. All variables were statistically significant at the 0.01 level. In most models, a 1°C rise in outdoor temperature is associated with a 0.22°C increase indoors, showing a strong relationship between the indoor and outdoor temperatures. Incorporating outdoor temperature lag in Model 5 increases the R-squared from 0.29 to 0.33, indicating a delayed influence of outdoor conditions on indoor temperature and improving the model’s explanatory power. The self-reported house condition shows positive coefficients in all models. This shows that houses with good conditions will tend to have higher indoor temperatures. Adding dwelling type in Model 2 raises explanatory power to 23%. High-rise buildings where occupants live on mid to high floors are approximately 0.47°C cooler than the reference cases, flat/apartment (high-rise on a low floor, or low-rise). Among tenure types, renting from a local authority/council has the largest coefficient, suggesting a significant influence on indoor temperature compared with other tenure categories.

**Table 4.** Regression results—independent variables and coefficients.

variable	Model 1	Model 2	Model 3	Model 4	Model 5
outdoor temperature	0.22	0.22	0.22	0.22	0.16
lagged outdoor temperature (6 h)	–	–	–	–	0.13
**house condition and socio economic**					
self-report house condition	0.18	0.10	0.40	0.39	0.42
EPC rating	0.07	0.03	0.17	0.15	0.19
IMD	0.43	0.65	0.13	0.19	0.01
**tenure**					
renting from a local authority/council	–	–	0.59	0.61	0.82
renting from a housing association/housing co-operative or charitable trust	–	–	−0.49	−0.47	−0.44
renting from a private landlord	–	–	−1.30	−1.27	−1.36
shared ownership	–	–	−0.79	−0.73	−0.76
**house type**					
Flat/apartment (high-rise, on a mid to high floor)	–	−0.47	–	−.06	−0.03
terraced house	–	0.24	–	0.16	0.07
constant	18.31	18.43	18.83	18.26	16.93
*R* ^2^	0.21	0.23	0.28	0.29	0.33

*Note*: Reference categories—tenure: homeowner with mortgage; house type: flat/apartment (high-rise on a low floor, or low-rise).

The IMD shows a positive correlation with indoor temperature, indicating that houses located in less deprived areas tend to have higher indoor temperatures. This finding is consistent with the earlier conclusion that better-maintained house conditions are associated with increased indoor temperatures, suggesting a potential link between socio-economic status, housing quality and thermal comfort.

## 6. Discussion

### (a) The curse of good condition

The findings of this study reveal a paradoxical relationship between building condition, socio-economic status and indoor temperature during the summer months. Buildings in good condition and those located in less deprived areas (as measured by the IMD) tend to exhibit higher indoor temperatures. Although better-maintained buildings and higher socio-economic status are typically associated with improved living standards, they may inadvertently contribute to overheating during summer, raising concerns about thermal comfort, health risks and energy consumption.

This ‘curse of good condition’ highlights a critical flaw in current building design and energy efficiency standards, which often prioritize winter performance at the expense of summer comfort. As global temperatures rise and heatwaves become more frequent, the unintended consequences of well-maintained buildings must be addressed to ensure year-round thermal comfort. This socio-economic paradox highlights the need for a more nuanced understanding of thermal comfort and energy efficiency. While better housing conditions and resources are generally desirable, they must be balanced with strategies to mitigate overheating and its associated risks.

The findings of this study have important implications for policymakers, architects and urban planners. Building codes and energy efficiency standards should be revised to address both heating and cooling needs. This includes promoting passive cooling strategies, such as natural ventilation, shading and reflective materials, to reduce indoor heat buildup. Raising awareness about the risks of indoor overheating and promoting adaptive behaviours, such as proper ventilation and shading, can help households manage indoor temperatures more effectively.

### (b) The invisible mini-heatwaves in domestic environments

The findings of this study complement current heatwave classification systems, which predominantly focus on outdoor meteorological conditions. The research exhibits the impact of extreme heat on indoor environments. Furthermore, with the varieties in building characteristics and socio-economic factors, the impact presents distinct differences. Through this analysis, it becomes evident that many mini-heatwaves occur within domestic environments, yet this phenomenon remains largely unaddressed in both research and policy.

Our analysis shows that indoor temperatures can differ significantly from outdoor temperatures owing to factors such as building insulation, ventilation and occupant behaviour. As a result, a heatwave defined by outdoor metrics may not accurately reflect the thermal conditions experienced indoors. Prolonged exposure to moderately high indoor temperatures, even if not classified as a heatwave outdoors, can have significant health effects. These mini-heatwaves within homes are often overlooked despite their potential to exacerbate heat-related illnesses.

This study reveals that many domestic environments experience frequent and prolonged periods of elevated indoor temperatures, which can be characterized as mini-heatwaves. These episodes are influenced by building characteristics and socio-economic factors.

The findings of this study highlight the need for a paradigm shift in how heatwaves are defined and addressed. Developing metrics that account for indoor thermal conditions, such as indoor temperature thresholds and duration, could provide a more accurate assessment of heat-related risks. Public health campaigns and heatwave preparedness plans should prioritize vulnerable populations and provide resources for improving indoor thermal comfort, such as subsidized cooling systems or heat relief programmes.

### (c) Limitation

While this study provides valuable insights, it is not without limitations. First, the reliance on self-reported data for house condition may introduce bias. Future research could incorporate objective measures of building quality, such as thermal imaging. Second, the study focuses on indoor temperature during summer; future work could explore seasonal variations and their effect on occupant health and wellbeing. Finally, the analysis is limited to a specific geographic context; expanding the study to other regions with different climatic and socio-economic conditions would enhance the generalizability of the findings.

## 7. Conclusion

The rapid acceleration of climate change is driving a steady increase in global temperatures and a rise in the frequency and intensity of extreme weather events. This presents an urgent and growing challenge for countries like the UK, which have traditionally enjoyed milder summer climates. This study leverages real-world sensor data to analyse indoor temperatures and thermal comfort levels in London homes, as well as their response to a heatwave during one of the UK’s hottest summers on record. Using the sensor collected data, the findings reveal that indoor thermal comfort levels frequently fall outside the acceptable range in most of the monitored homes. While the houses generally mitigated the impact of the outdoor heatwave to some degree, brief but intense heat spikes were observed in the majority of cases. In addition, the study highlights a strong correlation between indoor temperatures and socio-economic factors, alongside housing conditions. By utilizing sensor-based data, this research provides valuable insights into how residential buildings respond to extreme heat events, offering a foundation for future strategies to improve thermal resilience.

## Data Availability

The data are available at https://data.ubdc.ac.uk/datasets/sensor-safeguarded#repeated_field-1

## Footnotes

^1^

https://docs.smartcitizen.me/knowledge/
